# CTCF Mediates the Cis-Regulatory Hubs in Mouse Hearts

**DOI:** 10.3390/ijms26199834

**Published:** 2025-10-09

**Authors:** Mick Lee, Loïc Mangnier, Cory C. Padilla, Dominic Paul Lee, Wilson Tan, Wen Hao Zheng, Louis Hanqiang Gan, Ching Kit Chen, Yee Phong Lim, Rina Miao Qin Wang, Peter Yiqing Li, Yonglin Zhu, Steve Bilodeau, Alexandre Bureau, Roger Sik-Yin Foo, Chukwuemeka George Anene-Nzelu

**Affiliations:** 1 Cardiovascular Disease Translational Research Programme, Yong Loo Lin School of Medicine, National University of Singapore, Singapore 117599, Singapore; 2 Genome Institute of Singapore, A*STAR, Singapore 138672, Singapore; 3 Centre de Recherche CERVO, Québec, QC G1E 1T2, Canada; 4 Département de Médecine Sociale et Préventive, Université Laval, Québec, QC G1V 0A6, Canada; 5 Centre de Recherche en Données Massives de l’Université Laval, Québec, QC G1V 0A6, Canada; 6 Centre de Recherche du Centre Hospitalier Universitaire de Québec, Université Laval, Axe Oncologie, Québec, QC G1R 3S3, Canada; 7 Cantata Bio, 100 Enterprise Way Suite A-101, Scotts Valley, CA 95066, USA; 8 Department of Paediatrics, Yong Loo Lin School of Medicine, National University of Singapore, Singapore 119228, Singapore; 9 Khoo Teck Puat-National University Children’s Medical Institute, National University of Singapore, Singapore 119074, Singapore; 10 Montreal Heart Institute, Montreal, QC H1T 1C8, Canada; 11 Department of Medicine, University of Montreal, Montreal, QC H3T 1J4, Canada; 12 Centre de Recherche sur le Cancer de l’Université Laval, Québec, QC G1R 3S3, Canada; 13 Département de Biologie Moléculaire, Biochimie Médicale et Pathologie, Faculté de Médecine, Université Laval, Québec, QC G1V 0A6, Canada

**Keywords:** cis-regulatory hubs, enhancers, ctcf, activity-by-contact, mouse cardiomyocytes (CM)

## Abstract

The 3D chromatin architecture establishes a complex network of genes and regulatory elements necessary for transcriptomic regulation in development and disease. This network can be modeled by cis-regulatory hubs (CRH), which underscore the local functional interactions between enhancers and promoter regions and differ from other higher-order chromatin structures such as topologically associated domains (TAD). The activity-by-contact (ABC) model of enhancer–promoter regulation has been recently used in the identification of these CRHs, but little is known about the role of transcription factor CCTC binding factor (CTCF) on ABC scores and their consequent impact on CRHs. Here, we show that the loss of CTCF leads to a reorganization of the ABC-derived rankings of putative enhancers in the mouse heart, a global reduction in the total number of CRHs and an increase in the size of CRHs. Furthermore, CTCF loss leads to a higher percentage of CRHs that cross TAD boundaries. These results provide additional evidence to support the importance of CTCF in forming the regulatory networks necessary for gene regulation.

## 1. Introduction

Unraveling the role of the non-coding regulatory elements in gene regulation is important to gain mechanistic insight into key biological processes such as cell fate determination and disease pathogenesis [1]. Accordingly, tremendous effort has been deployed to annotate these regulatory elements in different tissues thanks to innovative sequencing techniques and computational tools [2,3,4]. These techniques include chromatin immunoprecipitation with sequencing (ChIP-seq), assay for transposase accessible chromatin with sequencing (ATAC-seq), and chromatin capture conformation with high-throughput sequencing (Hi-C) [5,6]. In addition, computational methods such as the activity-by-contact (ABC) algorithm [3] and other tools [7,8] have been developed to integrate these datasets and identify enhancer-gene (E-G) pairs required for development and disease [9,10,11].

Besides the direct identification of E-G pairs, other studies have demonstrated that enhancers and promoters interact dynamically within a network called cis-regulatory hubs (CRH), which allows for multiple regulatory connections [12,13,14,15]. These CRHs can be defined as 3D regulatory networks that constitute a complex organization of multi-loci connections of enhancers and promoters connected in 3D space within the nucleus. They highlight direct and indirect contact between genes and distal regulatory elements and can help predict target genes for disease-relevant non-coding single-nucleotide polymorphisms (SNPs) [14]. They are often bound by a cluster of transcription factors (TF) and have been implicated in gene co-regulation, lineage specification and disease development [13,14,16]. These hubs differ from other known 3D structures, such as genomic compartments, which constitute euchromatin or heterochromatin regions. They also differ from topologically associated domains (TADs) defined as units within which most enhancer–promoter interactions occur [17]. Although the identification of these CRHs is often performed solely through detailed analysis of 3D contacts from Hi-C data, [13,18,19,20,21], a recent study proposed building these CRHs using the ABC algorithm [14]. The ABC algorithm combines ATAC-seq, ChiP-seq and Hi-C to rank E-G pairs based on their regulatory impact [3,22]. Thus, in addition to identifying and ranking enhancers, the ABC algorithm can be used to generate a more functionally relevant CRH [14]. CRHs built using ABC were strongly enriched for disease-relevant genes and also helped explain disease heritability [14,23]. 

Given that the 3D genome architecture through HI-C is a key component of the ABC algorithm, which is used to construct the CRHs [14], it is of huge importance to understand the role of architectural proteins like CTCF in these CRHs. The CTCF protein, often called the “genome weaver,” plays a crucial function in the “folding” of the genome and brings enhancers into proximity to their distant target genes [17,24]. It thus plays key roles in regulating gene expression in various tissues by facilitating enhancer–promoter interactions and by preventing ectopic enhancer–promoter interactions [17]. We and others have shown the importance of CTCF in regulating cardiac chromatin architecture, gene expression and heart disease [25,26]. However, CTCF’s role in regulating CRHs has not been explored. Thus, this study aims to understand the role of CTCF in determining the composition and boundaries of ABC-derived CRH. In this study, using mouse cardiomyocytes as a model of terminally differentiated non-dividing cells, we employ the ABC model to identify top mouse cardiomyocyte gene-enhancers and then annotate the CRHs. We analyze the characteristics of the CRHs containing tissue-specific genes, showing that these genes are often found in multi-enhancer CRHs. We also show that in disease, there is a positive regulation of genes and enhancers within a CRH. Finally, we show that loss of CTCF leads to a merging of CRHs and the formation of new CRHs in the *Ctcf*-KO cells with resultant drastic change in the number and distribution of enhancers that cross the ABC threshold.

## 2. Results

### 2.1. Mouse Cardiomyocyte Enhancer Landscape Identified Through Activity-by-Contact Algorithm

**Figure 1:**
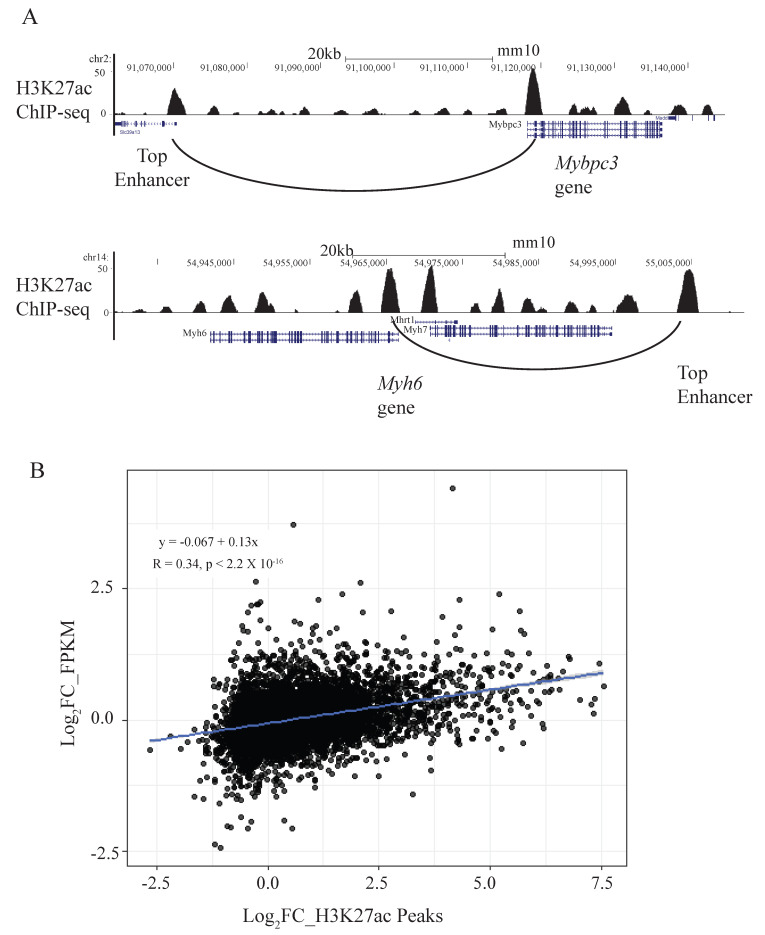
(**A**) USCS screenshot showing two examples of CM genes and their top ABC-linked enhancers; (**B**) Pearson correlation coefficient showing correlation between differentially expressed genes and differential enhancer peaks for ABC-linked enhancers in cardiac disease.

We performed ATAC-seq, H3K27ac ChiP-seq and HiChIP to generate the ABC scores for the enhancer-gene pairs in the control adult mouse cardiomyocytes (CM). The generation of the ABC scores is a first step in building the ABC-derived CRHs and unraveling the importance of CTCF in this process. Using an ABC cut-off of 0.01 [14], we identified ~7000 out of 156,988 putative distal regulatory regions marked by H3K27ac peaks and/or ATAC peaks in the adult mouse CM that crossed the threshold. This represents about 5% of all putative regulatory regions predicted to have strong regulatory effects on their target genes and is similar to what was observed in another study [3]. With regard to the enhancer-gene (E-G) pairs, globally there were 34,496 E-G interactions with an average of 4.19 connections per gene and 5.29 connections per enhancer in these healthy adult mouse CMs. Figure 1A shows examples of the top enhancers for 2 CM disease-relevant genes, *Mybpc3* and *Myh6*. The comprehensive list of ABC-linked enhancers and target genes can be found in Supplementary Table S1. A literature search confirmed that at least three of the identified enhancers in our study have been validated. These include the *Nppa/Nppb* super-enhancer located upstream of both genes, which has been shown to play a critical role in the stress gene response of *Nppa/Nppb* during pressure-overload-induced CM stress [25,27,28]. A separate enhancer for *Myh7* identified from the ABC scoring has also been validated previously in the mouse heart [29]. Deletion of this enhancer region led to the downregulation of *Myh7* in mouse hearts [29]. Taken together, these previous studies provide support for the validity of the ABC model.

For a better understanding of some of the features of highly ranked enhancers, we focused our attention on the top 1% and bottom 1% of enhancers based on the ABC score. The enhancers in the top 1% had scores of 0.9–1, while those in the bottom 1% had scores of 0.01. First, we analyzed the average distance of these enhancers to the identified target genes. As expected, enhancers with high scores were more likely to be closer to the target genes, with mean and median distances of 83 kb and 54 kb, respectively, as compared to the mean and median distances of the bottom 1% of enhancers, which were 950 kb and 699 kb, respectively (*p*-value < 0.01). Next, we performed a gene ontology (GO) analysis of the genes linked to these enhancers. The GO biological processes found in the genes which were linked to the top 1% of enhancers included terms such as striated muscle cell differentiation, regulation of blood circulation and actin cytoskeletal organization (Supplementary Table S2). These are processes that are important for CM function and include genes such as *Gata4*, *Mybpc2*, *Cox7a1* and *Myl3*. On the other hand, the GO for genes in the bottom 1% had generic terms like RNA processing, protein modification and cellular metabolic processes (Supplementary Table S2).

To provide further evidence that these ABC-linked enhancers regulate their putative target genes, we studied the correlation between changes in gene expression versus changes in peak height of the ABC-linked enhancers during pressure-overload-induced CM hypertrophy. For this analysis, we reanalyzed our previous work which detailed transcriptomic and epigenetic changes in a mouse model of heart failure through transverse aortic constriction [25]. Using RNA-seq and H3K27ac ChIP-seq datasets from that study, we found that changes in gene expression correlated modestly with changes in H3K27ac peak signals (Pearson correlation 0.34, *p* < 2.2 × 10^−16^) (Figure 1B). The correlation was stronger when focusing only on genes and enhancers with greater or less than a log_2_ 0.5-fold change after transverse aortic constriction (Supplementary Figure S1A) (Pearson correlation = 0.51, *p* < 2.2 × 10^−16^). This result shows that globally, upon external stimuli, gene expression is regulated in the same direction as its ABC-linked enhancers.

### 2.2. Loss of CTCF Leads to Changes in the Enhancer Interactome and Changes in the ABC Scores of Putative Enhancers

Next, to elucidate the role of CTCF in ABC scores and the regulation of the CRHs, we also applied the ABC algorithm on the *Ctcf*-KO mouse CMs. By analyzing the ABC scores and CRHs in *Ctcf*-KO mouse CMs, we could begin to infer the importance of CTCF in the ABC-derived CRH. From our analysis, we observed that 36,226 regulatory elements crossed the 0.01 threshold. This resulted in 71,080 E-G interactions with a similar number of expressed genes (~8000) (Supplementary Table S1) in control and *Ctcf*-KO samples. This led to an increase in the average number of connections per gene to 8.14 in *Ctcf*-KO samples. Of the 71,080 enhancer-gene pairs, only 20% (14,200 E-G pairs) were shared between control and *Ctcf*-KO mouse CMs. This increase in the number of enhancers with an ABC score greater than 0.01 could be due to increased interactions between genes and ectopic enhancers upon CTCF loss. Interestingly, we observed that while the highest-ranked ABC enhancer in the control had a score of 0.99, the highest-ranked enhancer in the *Ctcf*-KO sample had a score of 0.78 (scale of 0–1). Indeed, the average score in the control was 0.054 while the average score in the *Ctcf*-KO was 0.028 (*t*-test *p*-value < 2.2 × 10^−16^). This suggests that the loss of CTCF led to a gain in ectopic enhancers in the *Ctcf*-KO, with a redistribution of the ABC scores of the enhancers. 

### 2.3. Loss of CTCF Alters the Cis-Regulatory Hubs

**Figure 2:**
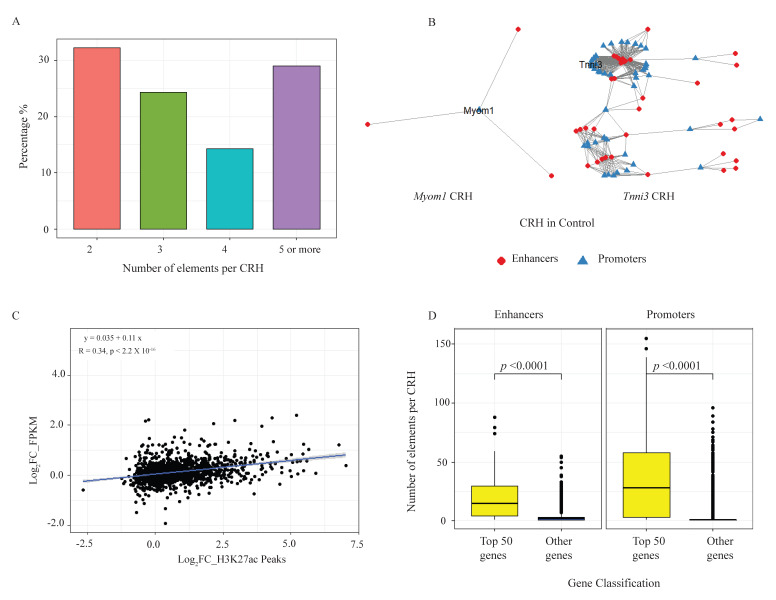
CRH in control cardiomyocytes. (**A**) Bar chart showing distribution of number of elements per CRH. (**B**) Examples of 2 CRHs containing CM genes Myom1 and Tnni3 in the control cardiomyocytes. (**C**) Pearson correlation coefficient between differential expression of genes and enhancer peaks contained within the same CRHs during CM stress. (**D**) Box plot showing characteristics of CRHs containing CM-specific genes. These CRHs tend to have more enhancers and promoters.

We integrated the ABC results to generate the CRHs, focusing first on the control samples. We identified 1522 hubs in these control cardiomyocytes with about 70% of the hubs containing fewer than 5 elements (Figure 2A). Figure 2B shows 2 examples of such hubs containing CM genes: *Myom1* is a gene involved in the formation of myofibrils [30] and is found in a simple hub. In contrast, *Tnni3*, a CM-specific sarcomeric gene, is found in a more complex hub. Consistent with the correlation between gene expression and ABC-linked enhancers in heart disease, we observed that genes and enhancers within the same hubs were positively correlated in the same direction of change during CM stress (Pearson correlation of 0.32 (*p* < 2.2 × 10^−16^)) (Figure 2C). This suggests a co-regulation of genes and enhancers found within the same hub upon external stimuli. We then ranked CM genes by FPKM and selected the top 50 genes, which were mostly CM-specific genes, to glean biological insights about the characteristics of CRHs that harbor these genes. Our data revealed that highly expressed CM genes were more likely to be in hubs with more regulatory elements than non-highly expressed genes (Figure 2D). Such multi-enhancer hubs have been proposed as a mechanism to buffer against external stresses and genetic perturbations and provide phenotypic robustness to disease-relevant genes [23,31].

**Figure 3:**
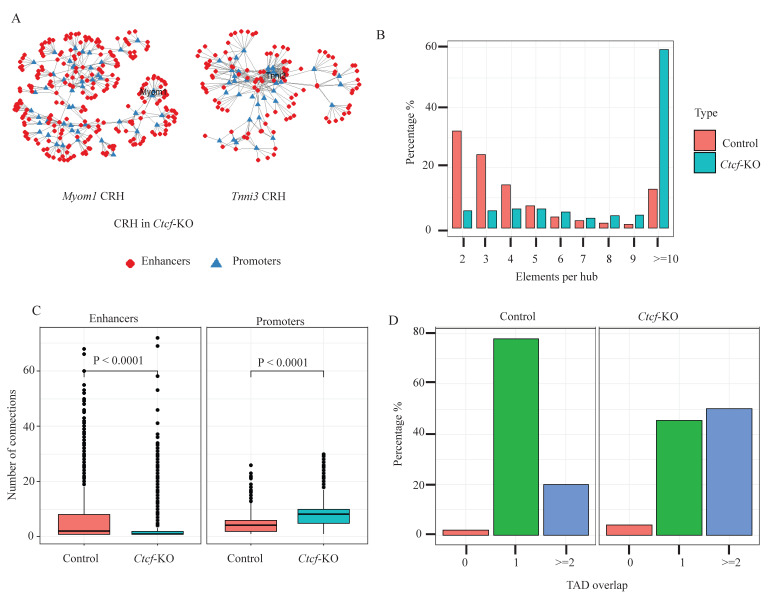
CRHs in the KO cells. (**A**) Examples of two CRHs containing the CM genes *Myom1* and *Tnni3* in the *Ctcf*-KO cardiomyocytes. Compared to the same CRHs in control, there is an increase in the number of elements in each hub. (**B**) Bar chart showing the distribution of elements per CRH in Control and *Ctcf*-KO. There is an increase in the percentage of CRHs with 10 or more elements from 15% in the control to 60% in the CTCF KO cardiomyocytes. (**C**) Bar chart showing the number of connections per gene promoters and per enhancers. There is an increase in the average number of connections per gene in the *Ctcf*-KO CMs. *p* < 0.0001. (**D**) Bar chart showing overlap of CRHs with TADs, 80% of TADs are contained in one TAD in the control, while only 40% of CRHs are contained within one TAD in the CTCF KO. In contrast, 20% of CRHs span two or more TADs in the control, while 50% of CRHs span two or more TADs in CTCF KO.

Next, we analyzed the CRHs in the *Ctcf*-KO CMs and found a marked reduction in the total number of CRHs, from 1522 in the control to 660 CRHs in the KO, accompanied by an increase in the number of elements per hub. Figure 3A shows an example of the same hubs containing two CM genes, shown in Figure 2A, showing an increase in the number of elements within each hub. Figure 3B shows the distribution of elements per hub, confirming a striking increase in the percentage of hubs with 10 or more elements. Associated with this increase in the percentage of complex hubs, there was an increase in the average number of connections per gene in the *Ctcf*-KO cells and a decrease in the number of connections per enhancer (Figure 3C, Supplementary Figure S1B). The decrease in the number of connections per enhancer was due to the overall increase in the number of enhancers that crossed the ABC threshold while maintaining the same number of expressed genes. Next, we asked if this reduction in the total number of CRHs in the *Ctcf*-KO was merely due to the merging of CRHs or the formation of new CRHs and observed both cases. The merging of CRHs was observed primarily in the simple hubs (2–3 elements per hub) located next to each other as 403 (82%) of the 492 simple hubs merged with 1 or more other hubs to form larger hubs. For the larger hubs, there was redistribution and formation of new hubs as genes gained new interactions and lost other interactions. To further confirm the importance of CTCF in the organization of these hubs, we compared CTCF binding sites in the control hubs vs. *Ctcf*-KO hubs. Our analysis showed an enrichment of CTCF in the control hubs (Fisher’s exact test odds ratio of 1.69, two-sided *p* < 2 × 10^−16^). Next, we analyzed the relationship between CRHs and TADs, since TAD boundaries are enriched for CTCF [25], and earlier studies have shown that CRHs are generally constrained within TAD boundaries [14]. Indeed, using the TADs in the control as a reference, our data showed that about 80% of CRHs in the control were contained within the same TAD, while only 20% spanned more than one TAD. In contrast, about 45% of CRHs in KO were contained within a TAD, while 50% of CRHs in the KO spanned more than one TAD (Figure 3D); furthermore, 5% were not within TAD boundaries, showing that the loss of CTCF leads to a reorganization of the CRHs with the formation of cross-TAD enhancer–promoter interactions.

Finally, we sought to identify if CRH changes were directly correlated to gene expression after CTCF loss. We have previously shown that CTCF KO leads to CM dysfunction and global transcriptomic changes after 2 weeks [25]; here, we wanted to gain deeper insight into how that related to CRH. Our analysis showed that despite the changes in the structure of CRHs, these changes were not directly reflected in gene expression. Indeed, there was no correlation between gain or loss of CRH connections and gene expression, suggesting that other secondary effects of the CTCF KO may have come into play to regulate the gene expression profile at 2 weeks when the RNA-seq was performed. This has been observed in other studies that showed that the direct effect of CTCF or Cohesin (another architectural protein) on RNA-seq is observed within a few hours of deletion [32,33]. As time progresses, other secondary factors come into play and obscure the direct effect of these proteins on gene expression. 

## 3. Discussion

Non-coding *cis*-regulatory elements (CRE) play crucial roles in development and disease and hold promise for next-generation therapeutic targets. While different studies by us and others have annotated these CREs and CRHs in different cellular models, the role of CTCF in ABC-derived CRHs has yet to be studied. First, our study applied the ABC algorithm to the mouse heart, generating, to the best of our knowledge, the first such ranked enhancer scores in mice hearts. Our findings confirm a positive correlation between genes and their ABC-linked enhancers in cardiac disease and affirm that genes and enhancers within the same hub are co-regulated during pressure-overload-induced CM hypertrophy. Integrating ABC to identify CRHs may thus represent another method to analyze the effect of regulatory SNPs on target genes, as the SNP may affect a target gene when they are in the same hub, even when there is no direct link through pair-wise E-G analysis [14]. Analysis of the CRHs also suggests that tissue-specific genes are more likely to be contained in hubs with high connectivity and rich in distal elements. This increased number of E-P networks provides redundancy and phenotypic robustness while guaranteeing increased transcriptional activity thanks to multi-enhancer networks.

Loss of CTCF affects the enhancer interactome, particularly impacting the H3K27ac interactions co-bound by CTCF. This leads to E-Ps that span larger distances and gain of new interactions. The loss of CTCF also leads to a re-organization of ABC-selected enhancers with a direct impact on the CRHs, resulting in fewer hubs but more elements per hub. This implies a crucial role for CTCF in determining which enhancers cross the ABC threshold and the organization of ABC-derived CRHs. While this study focused on the effect of loss of CTCF on overall CRH structure and its relationship with TAD boundaries, future studies will examine individual CRHs and their relationship to CTCF binding to ascertain how loss of CTCF can lead to local dysregulation of CRHs. Indeed, this finding has implications for mutations that affect local CTCF binding at regions that are not TAD boundaries, as it may lead to the merging of two or more CRHs and, thus, the establishment of an indirect connection between genes and enhancers within the newly formed hubs. In addition, given that the binding of CTCF to DNA is methylation-sensitive [34,35], this also supports findings that conditions that lead to DNA hypermethylation will reduce local or global CTCF binding [36]. This loss of CTCF binding will thus lead to the loss of insulation at TAD boundaries with a consequential gain of ectopic enhancers and reorganization of ABC enhancers [36]. 

## 4. Materials and Methods

### 4.1. Animal Experiments

Animal experiments were performed under a license approved by the Institutional Animal Care and Use Committee (National University of Singapore). *Ctcf*^flox/flox^ mice harboring LoxP sites flanking exons 3 and 12 of the *Ctcf* gene against a C57/bl6 strain background were used as previously published. AAV9-cTnt-eGFP and AAV9-cTnt-Cre-tdTomato vectors encoding codon improved Cre recombinase or eGFP under the control of cardiac troponin T promoter were used for the control and *Ctcf* knock-out, respectively [25]. Experiments were performed on adult 6- to 8-week-old mice. Cardiomyocytes were isolated from mouse hearts after 2 weeks of AAV9 injections for control and *Ctcf*-KO mice. Adeno-associated virus 9 (AAV9) virus for the delivery of Cre recombinase was generated as previously described [25].

### 4.2. H3k27ac HiChip Library Prep and Data Analysis

Mouse adult cardiomyocytes were isolated following previously published protocol [25]. MNase-based HiChIP assay was performed on 2 × 10^6^ isolated CMs using the Dovetail Genomics HiChIP MNase kit protocol [37]. Frozen cells were resuspended in 1× PBS and crosslinked with 3 mM DSG and 1% formaldehyde. Washed cells were digested with 0.75 µl MNase in 100 μl of nuclease digest buffer with MgCl_2_. Cells were lysed with 1× RIPA, and clarified lysate was used for ChIP. H3K27ac antibody (Abcam 04729, Cambridge, UK) was used to perform chromatin immunoprecipitation. The protein A/G bead pulldown, proximity ligation and libraries were prepared as described in the Dovetail protocol (Dovetail HiChIP MNase Kit, Cantata Bio, Scotts Valley, CA, USA). Libraries were sequenced on an Illumina Novaseq (Supplementary Table S3 shows the sequencing QC).

HiChIP paired-end reads were aligned with BWA MEM [38] (version 0.7.17r1198-dirty) with the -5SP flag enabled with an index containing only the canonical chromosomes of the mm10 genome (available from the UCSC genome). The resulting alignments were then parsed with pairtools (versions 0.3.0) with the following options –min-mapq 40 –walks-policy 5unque –max-inter-align gap 30 and the thre –chroms-path file corresponding to the size of the chromosomes used for the alignment index. Parse pairs were deduplicated and sorted with pairtools. Valid pairs were identified and through pairtools selected (pair type = ‘UU’, ‘UR’, ‘RU’, ‘uu’, ‘uU’ and ‘Uu’) and downsampled to the lowest number of valid pairs in a sample (126 million in LACZ) with pairtools sample. Contact matrices in .hic format were generated with the juicetools pre function (version 1.22.01).

FitHiChIP [39] (version 9.1) was used to identify significant interactions (“loops”) from the valid subsampled pairs at 10 kb resolution with the following settings: loop type = all-to-all, coverage = bias correction, merge redundant loops = Yes, background model = FithHiChIP(L), FDR < 0.1, minimum interaction size = 20 kb, maximum interaction size = 2 mb. Conditionally unique (meaning only occurring in LACZ or CRE) and shared loops were identified with bedtools pairToPair (version 2.28.0), requiring both loop anchors to overlap the same coordinates be flagged as shared. Loop size was assessed by performing a two-sided Wilcoxson’s rank-sum test on the distribution of loop ranges (distance between the two anchors). Loop anchors were then intersected with the LACZ CTCF binding sites to determine the proportion of loops resulting from CTCF presence. *p*-values for CTCF mediated loops were obtained through a two-sided Fischer’s Exact test. Aggregate Peak Analysis (APA) [32] was computed with juicetools APA function. Loop anchors were used to as the sites to aggregate over at 10 kb resolution. APA enrichment scores, loop center from the lower-left (LL) corner, are shown as both an APA score (ratio of the mean of center to mean of LL) and a Z-score. APA-normed output was used to plot the APA matrices.

H3K27ac HiChIP data were summarized and visualized with R; the package eulurr was used for the shared and unique loop Venn diagrams and ggplot2 for loop size and proportion of loops with CTCF binding site at the anchor. Additionally, ggplot2 was used to plot APA matrices with the geom_raster function, with the color scale set to the same min–max limits across all APA plots. At sites of interest, HiChIP loops files (in longrange format) were used to visualize loops along with ChIP-seq coverage and RNA-seq activity in the WashU Epigenome Browser (https://epigenomegateway.wustl.edu/). Supplementary Table S4 lists all the loops

### 4.3. RNA-Seq

RNA was extracted from 3 biological replicates of control and CTCF KO ventricular CMs (1 million cells each). The paired-end libraries were constructed using Tru-seq kits (Illumina, San Diego, CA, USA) and resulting libraries were sequenced on the Novaseq platform, generating 2 × 150 bp paired-end reads. The RNA reads were mapped to the mouse genome with Tophat version 2.0.11 with default parameters, and gene count was computed with htseq-count. Differential gene expression analysis was performed with EdgeR [40].

### 4.4. Chip-Seq Experiment

ChIP experiments on isolated CMs (1 million cells each) were performed as described previously [25]. Briefly, CMs were cross-linked with 1% formaldehyde for 10 min at room temperature and quenched with glycine (0.125 mol/L final concentration) [25]. Cells were then washed in ice-cold PBS and pelleted and lysed in FA lysis buffer (10 mmol/L Tris-HCl, pH 8.0, 0.25% Triton X-100, 10 mmol/L EDTA, 0.1 mol/L NaCl). To facilitate cell lysis, the cell pellet was passed through a 27.5-gauge needle gently 20 times. Nuclei were pelleted by centrifugation resuspended in sonication buffer, and chromatin was fragmented via sonication to an average size of 200 to 300 bp (Bioruptor Plus, Diagenode, Denville, NJ, USA). Chromatin was immunoprecipitated against H3K27ac (Abcam ab4729) or CTCF (EMD Millipore, catalog No. 07–729, Burlington, MA, USA) overnight. Antibody–chromatin complexes were pulled down with Protein G Dynabeads (Invitrogen, catalog No. 10003D, Carlsbad, CA, USA), washed with 0.1% SDS lysis buffer and eluted with elution buffer (1% SDS, 10 mmol/L EDTA, 50 mmol/L Tris-HCl, pH 8). After cross-link reversal (4 h of incubation at 65 °C) and proteinase K treatment, immunoprecipitated DNA was extracted. ChIP DNA was quantified by fluorometric quantification (Thermo Fisher Scientific, Waltham, MA, USA, Qubit dsDNA HS assay kit, catalog No. 32851). Library preparation was performed with the New England Biolabs Ultra II Kit (New England Biolabs, Ipswich, MA, USA) according to the manufacturer’s specifications and sequenced on the Illumina NextSeq High platform.

### 4.5. ATAC-Seq Experiment and Analysis

The ATAC-seq was performed for both control and *Ctcf* KO CMs according to the previously published Omni-ATAC protocol [41]. Briefly, 50,000 adult cardiomyocytes were pelleted at 500 g for 5 min. The cells were resuspended in 100 µL ATAC-resuspension buffer containing 0.5% NP40, 0.5 % Tween-20 and 0.01% Digitonin. The cells were left on ice for 5 min, after which 1 mL of cold ATAC-RSB containing 0.1% Tween 20 was added to wash out the lysis buffer. The nuclei were pelleted at 500 g for 5 min, the supernatant was carefully removed and the nuclei were resuspended in 50 µL transposition mixture containing 25 µL of 2x TD buffer, 16.5 µL of PBS, 5 µL nuclease free water, 0.5 µL of 1% digitonin, 0.5 µL of 10% Tween-20 and 2.5 µL of transposase (Illumina Tagment DNA enzyme 1, catalog number 20034198). The reaction was incubated at 37 °C for 30 min in a thermoshaker with 1000 RPM. After the reaction, DNA was extracted using the NEB Monarch^®^ PCR & DNA cleanup kit (catalog number T1030L, New England Biolabs), PCR was performed using the Illumina/Nextera primers after which Ampure XP beads (Beckman Coulter, Indianapolis, IN, USA) were used for library cleanup. The resulting library was sequenced on Nextseq. 

### 4.6. ChIP-Seq and ATAC-Seq Data Analysis

For both H3K27ac ChIP-Seq and ATAC-Seq, fastq files were aligned to the mm10 reference genome using the Burrows-Wheeler alignment tool in bwa software v0.7.19 [38]. The corresponding bam files were obtained using samtools v1.22.1 [42], while peak calling was performed with macS2 software v2.2.9.1 [43]. All peaks with a q-value less or equal to 0.1 were considered as statistically significant and used in the activity-by-contact score [3] for CRH construction [14]. Supplementary Table S5 lists H3K27ac peaks in Sham and TAC-treated mouse cardiomyocytes, as well as those in control vs. *Ctcf*-KO cardiomyocytes.

### 4.7. ABC-Score and CRH Analysis

The ABC model defines active enhancers based on a quantitative score of DNAse or ATAC-seq, H3K27ac and normalized Hi-C contact number [3]. This score is computed relative to a background activity over a 5 Mb window around a candidate element. Then, we set the threshold to 0.01, beyond which a candidate element is considered as a distal element. As an extension of the ABC score, CRHs [14] were defined as bipartite networks between promoters and distal elements (igraph R package) [44]. TADs were called using the directionality index as previously described. Pearson correlation analysis was performed using an R package (v2.1.4).

## 5. Conclusions

Our findings demonstrate that CTCF plays a critical role in determining the regulatory strength of enhancers as well as the membership and organization of the CRHs. Given that the CRHs play a role in disease and cell-type-specific gene expression, processes that affect CTCF expression and genomic binding will lead to a disorganization of CRHs and a subsequent effect on transcriptional regulation upon external stimuli. Although we did not find a correlation between the reorganization of CRHs and gene expression upon the loss of CTCF, this was mostly due to other secondary effects in the regulation of gene expression. However, we believe that our findings reveal important principles of the role of CTCF in regulating CRHs and ABC scores. A major limitation of our study is that the resolution of 10 kb for our HiChIP is not optimal, as each 10 kb window may contain various enhancers, and hence our analysis may miss out on some enhancer-gene interactions. In addition, as the various components of the ABC analysis, namely ATAC, ChIP and HiC, may have technical variations, an independent ABC analysis would be valuable to confirm our findings. However, we believe that our study forms the foundation for future analysis on the role of CTCF in ABC enhancer scores and as such the relative contribution of enhancers to each gene. Our future studies will delve deeper into these questions.

## Data Availability

The raw data supporting the conclusion of this article has been deposited in a public repository.

## Supplementary Materials

The following supporting information can be downloaded at: https://www.mdpi.com/article/10.3390/ijms26199834/s1.
