# Supplementary figures and images for: CTCF Mediates the Cis-Regulatory Hubs in Mouse Hearts
Source: Int J Mol Sci. 2025 Oct 9;26(19):9834. doi: 10.3390/ijms26199834 (PMC12524426; doi:10.3390/ijms26199834)

A

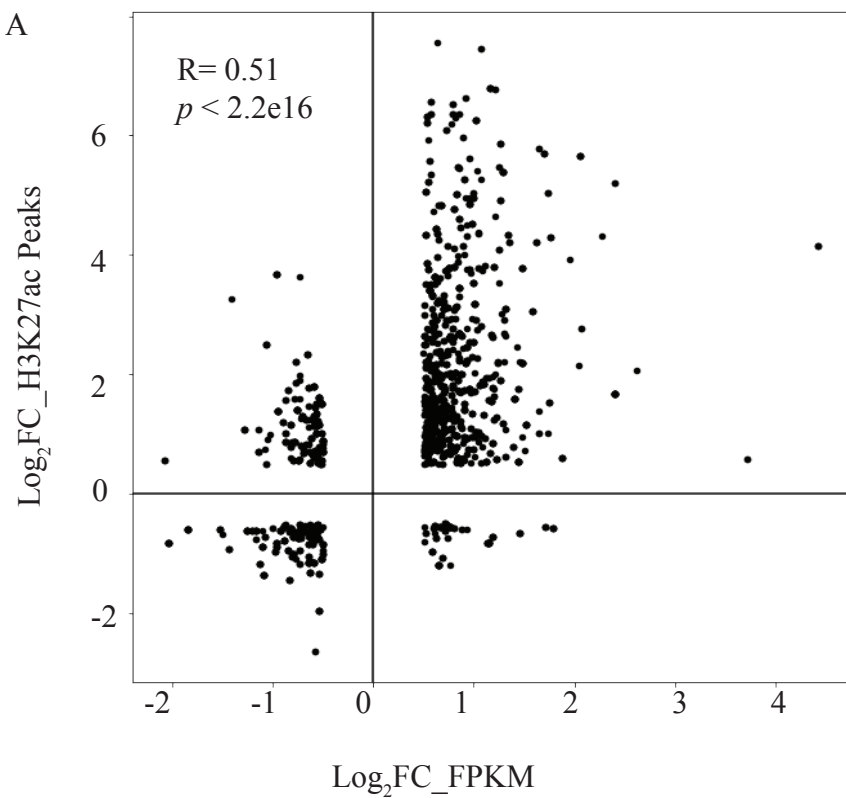

B

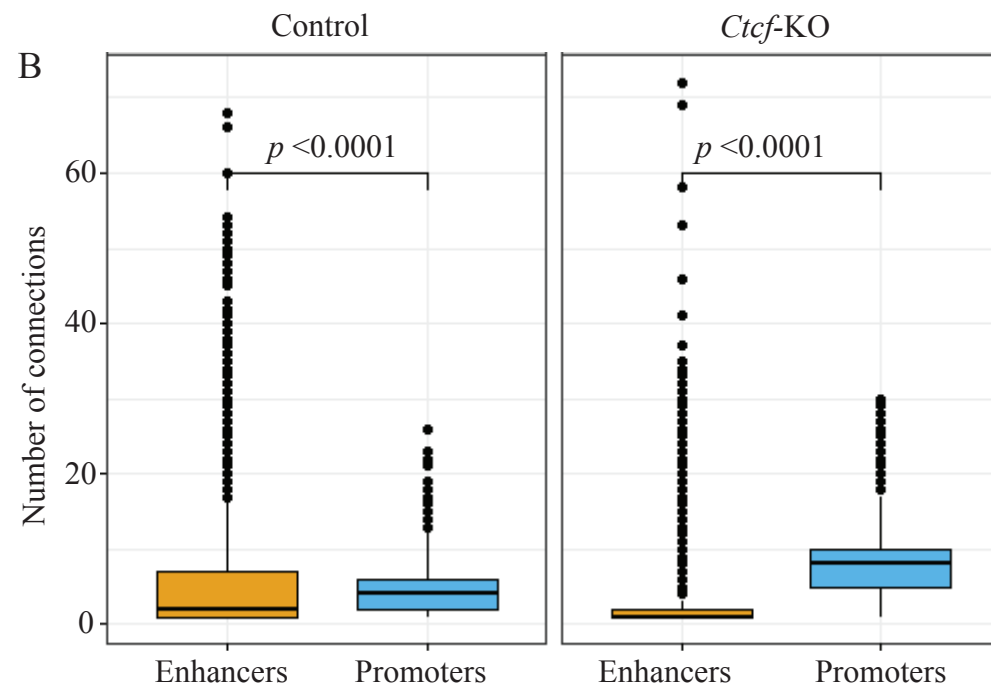

Supplement: Supplementary file 1 [file ijms-26-09834-s001.zip › for zip/Supplementary Figure 1 updated.pdf]
